# microRNA from brush biopsy to characterize oral squamous cell carcinoma epithelium

**DOI:** 10.1002/cam4.951

**Published:** 2016-12-18

**Authors:** Yalu Zhou, Antonia Kolokythas, Joel L. Schwartz, Joel B. Epstein, Guy R. Adami

**Affiliations:** ^1^ Department of Oral Medicine and Oral DiagnosticsCenter for Molecular Biology of Oral DiseasesUniversity of Illinois at Chicago801 South Paulina StreetChicagoIllinois60610; ^2^ Arphion Ltd2242 W Harrison StreetChicagoIllinois60612; ^3^ Department of Oral and Maxillofacial SurgeryCollege of DentistryUniversity of Illinois at Chicago801 South Paulina StreetChicagoIllinois60610; ^4^ Cancer Dentistry ProgramSamuel Oschin Comprehensive Cancer CenterCedars‐Sinai Medical CenterLos AngelesCalifornia

**Keywords:** Brush biopsy, head and neck cancer, miRNA, the Cancer Genome Atlas

## Abstract

Few cancers are diagnosed based on RNA expression signatures. Oral squamous cell carcinoma (OSCC) is no exception; it is currently diagnosed by scalpel biopsy followed by histopathology. This study sought to identify oral tumor epithelial microRNA (miRNA) expression changes to determine if these changes could be used to diagnose the disease noninvasively. Analysis of miRNA profiles from surgically obtained OSCC tissue, collected under highly standardized conditions for The Cancer Genome Atlas, was done to determine the potential accuracy in differentiating tumor from normal mucosal tissue. Even when using small 20 subject datasets, classification based on miRNA was 90 to 100% accurate. To develop a noninvasive classifier for OSSC, analysis of brush biopsy miRNA was done and showed 87% accuracy in differentiating tumor from normal epithelium when using RT‐qPCR or miRNAseq to measure miRNAs. An extensive overlap was seen in differentially expressed miRNAs in oral squamous cell carcinoma epithelium obtained using brush biopsy and those reported in saliva and serum of oral squamous cell carcinoma patients in several studies. This suggested that nonselective release of these miRNAs into body fluids from tumor epithelium was largely responsible for the changes in levels in these fluids seen with this disease. Using a variation in mirRPath we identified the KEGG pathway of neurotrophin signaling as a target of these miRNAs disregulated in tumor epithelium. This highlights the utility of brush biopsy of oral mucosa to allow simple acquisition of cancer relevant miRNA information from tumor epithelium.

## Introduction

Oral cancer will be diagnosed in approximately 30,000 people in the United States this year, and close to 400,000 in the world 1. In large regions of Southeast Asia it is the second most‐diagnosed cancer. The disease is typically found on the surface of the tongue or gingiva, but can occur anywhere in the oral mucosa. Over 90% of oral cancers are oral squamous cell carcinoma (OSCC). While oral lesions are easily detectable by dentists, only a small percentage will be OSCC. The initial diagnosis requires scalpel biopsy by an oral surgeon, followed by histopathology examination. Because the majority go undiagnosed till the late stages, the disease often has a poor prognosis with average survival times <5 years 2, 3. Much effort has gone into improving lesion detection and diagnosis and one way is to remove the need for scalpel biopsy. This has been attempted using special scanning devices based on either infrared light or fluorescence 2, 3. These approaches have the possibility of easing patient concerns about surgical biopsy while also potentially making it possible to detect and diagnose in one step. Others have used gene‐based methods to determine changes in the oral mucosa indicative of cancer. First with mRNA, and then miRNA, RNA signatures for OSCC have been developed using surgically obtained tissue 4, 5, 6, 7. Results from these surgical specimens, which contain a variable mixture of epithelium and tumor stroma, produce different results between studies 8, 9. A second approach has looked for markers of OSCC in body fluids, such as blood or saliva, with interesting, but, likely due to low RNA concentrations, variable results 10, 11, 12, 13, 14, 15, 16, 17, 18. The limited follow‐up on published RNA classifiers for OSCC combined with the lack of standardized sample collection methods for RNA‐based detection and diagnosis has slowed validation for clinical purposes.

The question remains whether improvements in sensitivity and specificity for consistent detection of critical epithelial change will ever allow identification of an RNA signature for OSCC, even under conditions where tissues are dissected and prepared uniformly. The release of The Cancer Genome Atlas (TCGA) dataset of head and neck cancers allows us to address this question as the samples were harvested surgically with uniform methods with reports of levels of normal tissue and stroma in each OSCC sample prior to RNA purification 19, and there was sufficient number of samples to allow extensive validation. At the same time this work reinforced a previous finding that OSCCs fall into discrete groups based on mRNA and miRNA expression 19, 20, 21. Because of that the variety of RNA expression associated with OSCC may be too complex to allow the creation of a single RNA signature associated with the disease.

In this study, we first tested if it was possible to develop a miRNA‐based classifier of OSCC under the highly standardized conditions of a single large study with uniform sample preparation 19. We evaluated the ability of miRNA expression profiles obtained from a small subgroup of OSCC and controls from the TCGA dataset to produce a validated miRNA signature specific for OSCC. We then built on work that showed mRNA can be harvested by brush biopsy form oral mucosa and individual mRNA species measured by RT‐PCR 8, 22, 23, 24, 25 though quality was variable 15, 25, 26, 27. We focused on miRNA due to an earlier observation that RNA from brush biopsy gives an accurate measure of oral epithelial miRNA 26. We used noninvasive brush oral biopsy with the goal of developing noninvasive OSCC detection and diagnosis 2, 28. We used two approaches, miRNAseq and qRT‐PCR, to measure change in miRNA levels in brush biopsy samples from both tumor epithelium and normal epithelium. We took advantage of the fact that our samples are almost exclusively epithelium to identify miRNAs associated with OSCC and used a modified approach to examine “in silico” molecular pathways targeted by these OSCC epithelial miRNAs.

## Materials and Methods

### Sample Collection

Brush biopsy samples were collected as described earlier from patients in the Oral and Maxillofacial Surgery Clinic in the University of Illinois Medical Center just prior to diagnostic biopsy or extirpative surgery (Table S1) 29. Some samples have been described earlier 29, newly acquired OSCC samples are described in the Table S2. Control samples were from subjects who on clinical examination revealed no suspicious lesions, the majority but not all were followed up over a year. The protocol used to obtain samples from patients after informed consent was approved by the Office for the Protection of Research Subjects of the University of Illinois at Chicago, the local Institutional Review Board.

### Histopathological confirmation

Of the 23 subjects with OSCC, all were diagnosed by surgical biopsy followed by histopathology and then this was confirmed post surgery. For 17 of the samples, the slides were available and these were reviewed by a third pathologist who confirmed the diagnosis as OSCC; this included the three cases that had equivocal miRNA‐based identification, OSCC305K, OSCC355, and OSCC413. OSCC329, 357 42910, 383, 583, 587, and 589 were examined by two pathologists.

### RNA purification

As described earlier, we used RNeasy chromatography (Qiagen, Germantown, MD) to remove mRNA followed by ethanol addition and RNeasy MinElute chromatography (Qiagen) to bind then elute small RNAs, including mature miRNA 29.

### miRNA quantification by miRNAseq

Small RNA libraries were constructed from 100 ng small RNA and sequenced at the W. M. Keck Center for Comparative and Functional Genomics at the University of Illinois at Urbana‐Champaign under the direction of Alvaro Hernandez. Small RNA libraries were constructed from the RNA samples using the TruSeq Small RNA Sample Preparation Kit (Illumina, San Diego, CA) with modifications as described earlier 30 with size selection of pooled barcoded libraries post‐PCR amplification so to enrich for small RNAs 18–50 nt in length. The final libraries were quantified by Qubit (Life Technologies, Carlsbad, CA) and the average size was determined on an Agilent Bioanalyzer High Sensitivity DNA chip (Agilent Technologies, Santa Clara, CA). The libraries were sequenced from one end of the molecule to a total read length of 50 nt on the Illumina HiSeq2500. The raw.bcl files were converted into demultiplexed FASTQ files with Casava 1.8.2 (Illumina).

### miRNAseq data analysis

Sequence files were received as FASTQ files, which were imported into Galaxy where adaptors were trimmed and quality assessed. Sequences of 17 bases and more were preserved and the collapse program in Galaxy was used to combine and count like sequences. FASTA files were uploaded in sRNAbench 1.0 which is now part of RNAtools http://bioinfo5.ugr.es/srnatoolbox/srnabench/. 30, 31. We used the h19 genome build miRNA library and selected 17 as seed length for alignment. The output Excel files of read counts for each known miRNA for each sample were combined into one and postnormalization was imported into BRB‐Array Tools to allow class comparison of differentially expressed miRNAs allowing up to 60% of reads for each miRNA to be zero 26, 32. This program was used to generate heat maps that allow a visualization of coordinately differentially expressed miRNAs. Tumor samples are more frequently contaminated with blood, which provide an excess of RBC markers, miR‐451a, miR‐144‐3p, and miR‐144‐5p, that for the purpose of this study are ignored. The class prediction tools of the site were used to test the seven different class prediction algorithms and their ability to generate, using leave‐one‐out cross‐validation, a classifier to differentiate the two samples types and then test the composite classifier on the individual samples using leave‐one‐out cross‐validation. Optimization of the cut‐off for significance levels for differences in miRNA quantities between classes was embedded in classifier generation so to avoid bias.

### miRNA quantification by qRT‐PCR

Most tumor samples were analyzed in a previous study by RT‐qPCR as described 29. Ten nanograms RNA from additional tumor and most normal samples was reverse transcribed in 5 *μ*L reactions using the miRCURY LNA Universal RT microRNA PCR, Polyadenylation, and cDNA synthesis kit (Exiqon, Woburn, MA). cDNA was diluted 20‐fold and assayed in 10 *μ*L PCR reactions according to the protocol for miRCURY LNA Universal RT microRNA PCR against a panel of four miRNAs and a spike‐in control for cDNA synthesis. When duplicate samples were available from a single lesion, the higher yield sample was subjected to a scaled‐up cDNA synthesis and was assayed by RT‐qPCR on the microRNA Ready‐to‐Use PCR, Human panel I (Exiqon), which includes 372 miRNA primer sets. The amplification was performed in an Applied Biosystems Viia 7 RT‐qPCR System (Life Technologies) in 384‐well plates. The amplification curves were analyzed for Ct values using the built‐in software, with a single baseline and threshold set manually for each plate.

Analysis of RT‐qPCR miRNA generated data was done as described for miRNAseq except the data were already log transformed prior to analysis with the BRB‐Array Tools program. Rank product analysis was done to identify likely differentially expressed miRNAs as described 32, 33 Only miRNAs detectable in over 60% of samples were examined.

### Expression data normalization

For RT‐PCR generated expression levels, Excel was used to normalize expression to a reference sample based on comparison to the value of 40 miRNAs in the panel that were found to be present in every sample. For miRNAseq the same methodology was used to normalize expression among the expression values except an overlapping but different set of consistently detected 50 miRNAs was used to determine the normalization factor.

### Prediction of miRNA targets and their functional analysis

Potential miRNA targets were identified using DIANA‐miRPath v3.0 (http://www.microrna.gr/miRPathv3). Only experimentally validated mRNA targets were selected, using Tarbase v7.0 (http://microrna.gr/tarbase). For functional annotation of potential targets we used Kyoto Encyclopedia of Genes and Genomes (KEGG) pathways term enrichment analysis using the computational tool miRPath to identify a list of pathways that show higher levels of representation than that expected when just examining random mRNAs. The preliminary miRNA pathway analysis shown in Table S8 revealed 25 KEGG pathways were significantly enriched (*P *< 1 × 10^−6^) in case of the differentially expressed miRNAs. Unfortunately, as demonstrated by Godard and van Eyll, standard miRNA target pathway analysis can lead to a high level of false positives, because, among other reasons, the known databases of all miRNA targets are overweighted with pathways involved in carcinogenesis, cell cycle regulation, etc. 34. To counter this, and other problems, we did this same analysis with 20 random sets of 11 miRNAs known to be expressed by human cells. This repetitive random miRNA target pathway analyses run as negative controls revealed many of the same targeted pathways in multiple queries (Table S9). One pathway targeted by the 11 OSCC miRNAs was found that did not appear in any of the negative controls. This conservative approach may have missed other bona fide targeted pathways.

## Results

### Differentiating normal and OSCC tissue based on miNRA profiles of surgically obtained oral tissue using TCGA dataset

**Figure 1:**
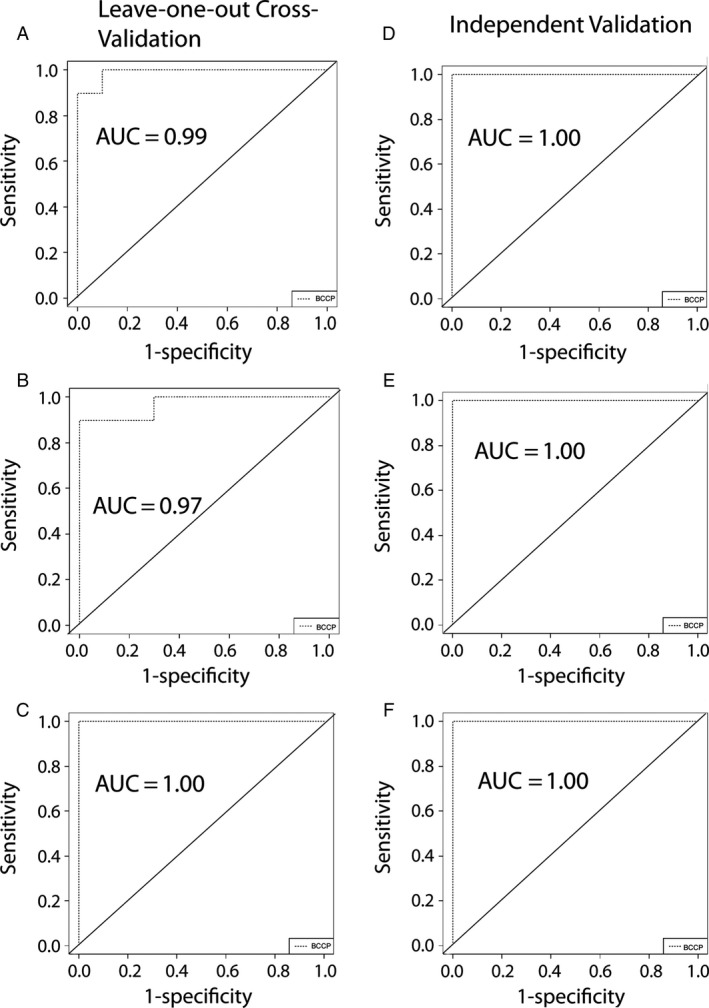
Three cohorts of miRNA profiles of 10 control and 10 OSCC samples were created from TCGA dataset of oral tumors and used to train 3 distinct OSCC classifiers. (A) Receiver operating characteristic curve of the Bayesian Compound Covariate based classifier for OSCC versus normal tested using leave‐one‐out cross‐validation cohort 1. (B) Receiver operating characteristic curve of the Bayesian Compound Covariate based classifier for OSCC versus normal tested using leave‐one‐out cross‐validation cohort 2. (C) Receiver operating characteristic curve of the Bayesian Compound Covariate based classifier for OSCC versus normal tested using Leave‐one‐out cross‐validation cohort 3. (D) The classifier generated on cohort 1 produced the diagrammed receiver operating characteristic curve when tested on 10 controls plus 20 OSCCs. (E) Same as (D) except the cohort 2 trained classifier was tested. (F) Same as (D) except the cohort 3 trained classifier was tested.

To first determine the practicality of establishing a miRNA‐based signature for OSCC, we turned to the TCGA dataset of miRNAseq‐based expression profiles of well over 500 miRNAs of surgically obtained tissue from 330 OSCCs. Thirty additional samples from normal appearing mucosa from a subset of patients were also included. Within the BRB‐Array Tools program seven different algorithms were used with leave‐one‐out cross‐validation to develop seven classifiers to differentiate tumor versus normal control with roughly similar accuracy (Fig. 1). miRNA profiles from just 10 tumor samples and 10 controls samples of the TCGA dataset were used to train OSCC classifiers. This was done three times with separate sets. Leave‐one‐out cross‐validation revealed on average, 95%, 90%, and 100% accuracy for all seven algorithms, though only the Bayesian Compound Covariate Classifier results are shown. The OSCC classifiers all showed 100% accuracy in identifying malignancy during external validation using independent subsets of 20 OSCC and 10 control samples from the same TCGA dataset. We were able to distinguish OSCC from normal surgical samples despite the fact that different subtypes of OSCC show different mRNA and miRNA profiles 19, 20, 21, 35. Class comparison revealed 36% and 41% overlap in the miRNAs that made up each classifier. Tables S3, S4, and S5 provides the list of miRNAs used in the Bayesian Compound Covariate predictors.

### Differentiating normal and OSCC cellular miRNA based on miRNA profiles of brush biopsy obtained RNA measured using next‐generation sequencing

**Figure 2:**
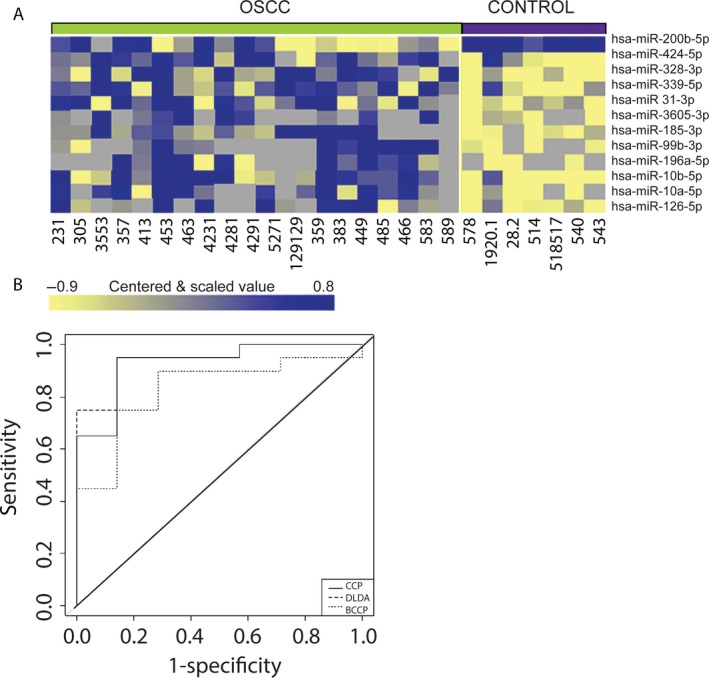
miRNAseq‐based miRNA profiles of brush biopsy samples and OSCC prediction. (A) Clustered heatmap of significantly differentially expressed miRNAs; samples grouped by class, tumor versus normal epithelium. (B) Receiver operating characteristic curve shows performance of the classifier generated using three different algorithms Compound Covariate Predictor, (CCP) Linear Diagonal Discriminant Analysis (LDDA), and Bayesian Compound Covariate Predictor and tested using leave‐one‐out cross‐validation.

While both mRNA and miRNA can be obtained from oral mucosa with a biopsy brush there has been some evidence that quality of mRNA can be variable 8. It has been our observation that some biopsy samples contain insufficient mRNA to allow measurement even with RT‐PCR, especially normal samples (data not shown). For that reason we focused on miRNA, which has shown consistent quality in the past 29, 36. To determine if RNA from brush biopsy of OSCC could be used to distinguish this disease from normal, we obtained 22 samples from OSCC patients with histopathology diagnosed OSCC. Fourteen controls were from separate subjects who showed no visible mucosal disorder. Of these, 20 tumor samples and seven control samples showed sufficient yield to allow sequencing of miRNA using miRNAseq methodology. Class comparison using BRB‐Array Tools showed differential expression of 13 miRNAs at a cutoff of False Discovery Rate (FDR) of 0.10 (Fig. 2A). Seven different algorithms were developed and tested using leave‐one‐out cross‐validation, which revealed 87% accuracy on average in differentiating tumor versus normal control. Receiver operating characteristic curves for three representative types of OSCC classifiers are shown in Figure 2B. Two samples in Figure 2 were consistently misclassified and include one tumor and one control regardless of the methodology used to formulate the classifier. The list of miRNAs used to formulate classifiers is provided in Table S6.

### Differentiating normal and OSCC cellular miRNA based on miRNA profiles of brush biopsy obtained RNA measured using qRT‐PCR‐based arrays

**Figure 3:**
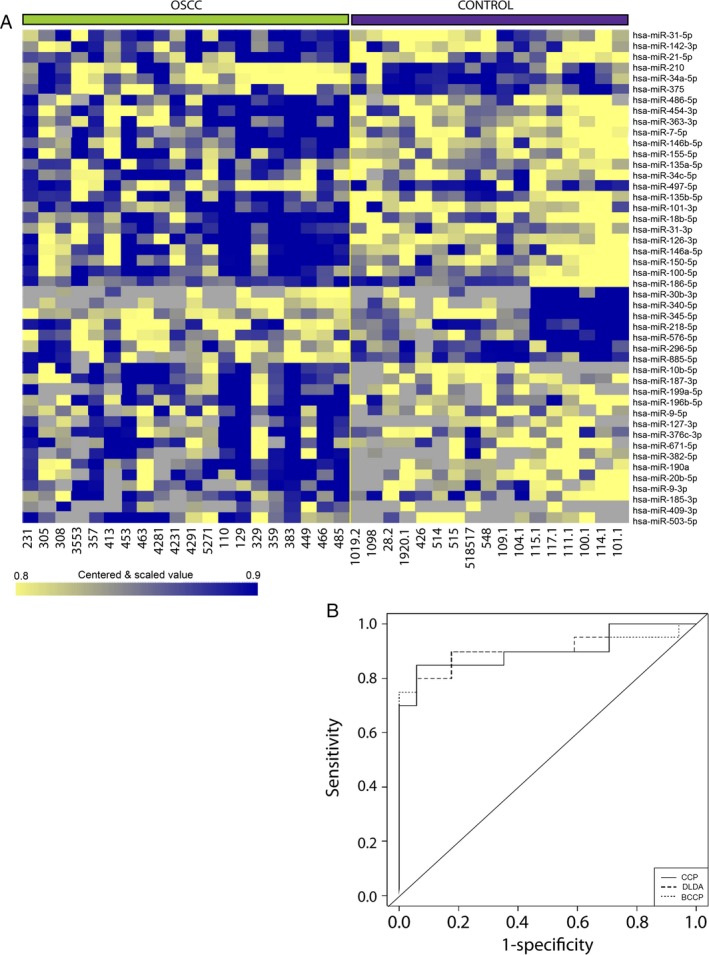
qRT‐PCR‐based miRNA profiles of brush biopsy samples and OSCC prediction. (A) Clustered heatmap of significantly differentially expressed miRNAs; samples grouped by class, tumor versus normal epithelium. (B) Receiver operating characteristic curve shows performance of the classifier generated using three different algorithms Compound Covariate Predictor, (CCP) Linear Diagonal Discriminant Analysis (LDDA) and Bayesian Compound Covariate Predictor and tested using leave‐one‐out cross‐validation. OSCC, Oral squamous cell carcinoma.

We originally chose miRNAseq to profile mature miRNA expression because methodology allowed testing more targets and was not limited to known miRNA sequences. However, the method used proved relatively insensitive and nine of 36 samples did not provide sufficient material to allow gene expression analysis. For that reason a qRT‐PCR platform that measures mature miRNA levels in the brush biopsy samples using LNA base primer probes was used. This more sensitive method produced miRNA expression profiles of 20 brush biopsy samples from OSCCs and 17 control samples from normal mucosa of nine smokers and eight nonsmokers. A list of 46 miRNAs showed differential expression at a FDR of 0.10, a result diagrammed in the heat map in Figure 3A. Again, simultaneous testing of multiple algorithms using leave‐one‐out cross‐validation provided a set of classifiers that on average distinguished tumor from normal with 87% accuracy among six different types of OSCC RNA‐based classifiers generated with the BRB‐Array Tools analysis. Receiver operating characteristic curves for three of these classifiers are shown in Figure 3B. The list of miRNAs used to formulate classifiers is shown in Table S7.

### Comparing miRNAs differentially expressed in normal and OSCC samples from brush biopsy and surgery

Given the substantial number of miRNAS described in this study as being differentially expressed in OSCC we compared our findings to that of the oral tumors and controls of the TCGA project, which examined a combination of tumor and stromal tissue in surgical samples. We found some overlap. Of the 46 miRNAs shown to be differentially expressed at least twofold in the brush biopsy samples from OSCC versus controls, 17 showed similar differential expression in the TCGA study of surgical samples at *P* < 0.05 with no correction for multiple testing. Direct comparison between the two datasets is made difficult by the lack of unambiguous labeling of the miRNAs from the TCGA dataset.

### KEGG pathways targeted by OSCC miRNAs

**Figure 4:**
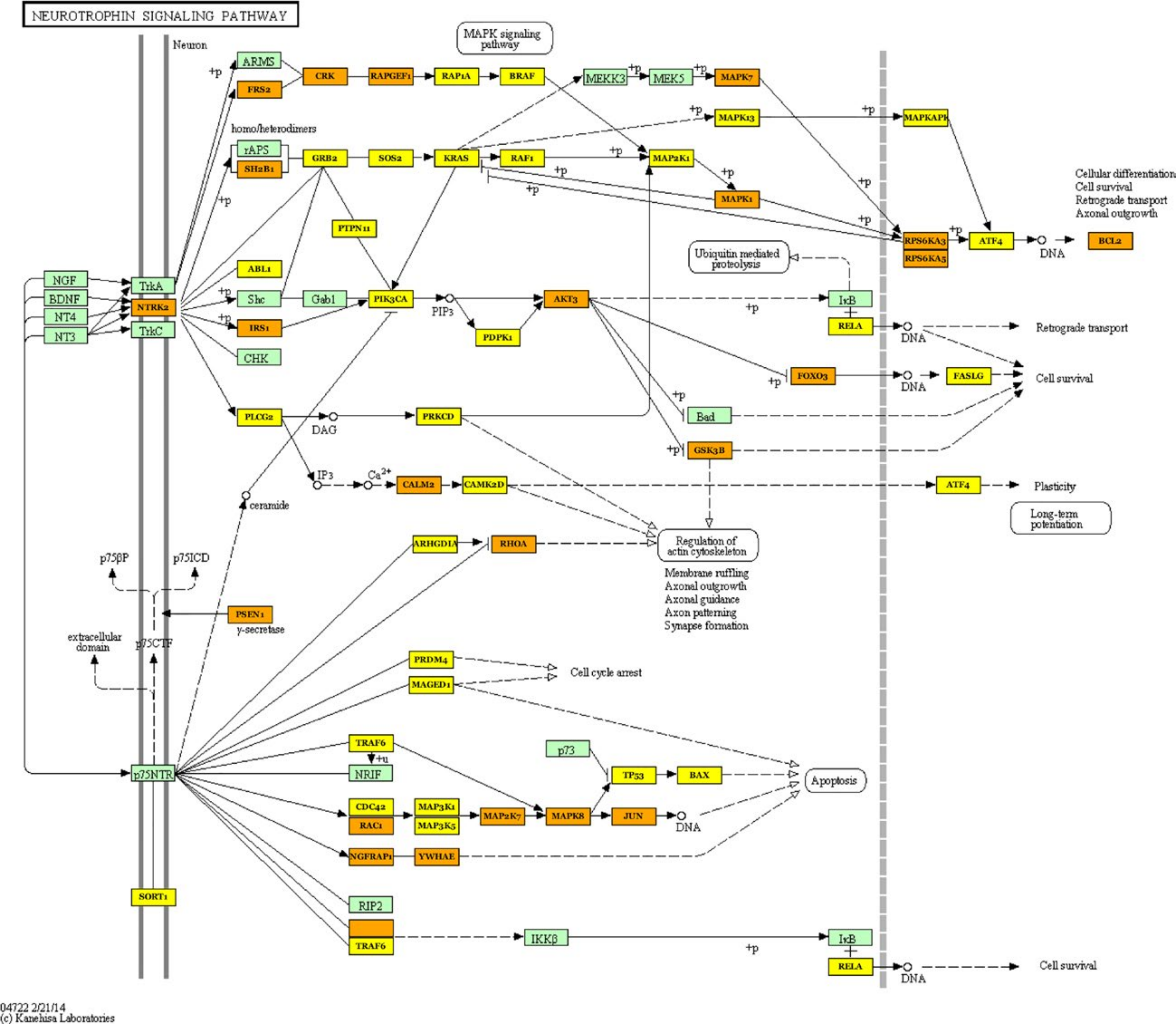
miRNAs enriched with OSCC target mRNAs of the neurotrophin signaling pathway. Eleven miRNAs were identified as enriched by at least 2× and FDR of <0.007 in OSCC versus normal epithelium. mRNAs targeted by one of these miRNAs are in yellow rectangles, whereas those targeted by at least two miRNAs are in gold. An examination of expression of the pathway mRNAs in the TCGA dataset revealed 5 that were decreased to at least 0.5× and one that was unexpectedly increased to 2x. Not all are shown in this simplified pathway diagram but all are identified in table 1. OSCC, Oral squamous cell carcinoma.

We examined the targets of differentially regulated miRNAs, for enriched functional pathways using DIANA‐miRPath v.2 37. miRPath allows the identification of molecular pathways in KEGG linked to changes in miRNA expression. We used this program to identify using DIANA‐TarBase v. 7.0 experimentally verified targeted mRNAs of the 11 microRNAs which showed at least FDR < 0.007 differential expression with OSCC and 2× or more change in expression, hsa‐miR‐486‐5p, hsa‐miR‐7‐5p, hsa‐miR‐146b‐5p, hsa‐miR‐101‐3p, hsa‐miR‐18b‐5p, hsa‐miR‐10b‐5p, hsa‐miR‐21‐5p, hsa‐miR‐190a, hsa‐miR‐20b‐5p, hsa‐miR‐126‐3p, and hsa‐miR‐31‐5p (Table S7). MiRPath allowed the identification of KEGG pathways linked to the mRNA targets. After using a new method to eliminate KEGG molecular pathways that were likely to be erroneously identified, we found one pathway, that of neurotrophin signaling, with a high probability of showing change in regulation with OSCC due to these miRNAs (see Methods and Materials, Tables S8 and S9, Fig. 4). As the mRNA quality was variable in the brush biopsy samples we used the TCGA database of mRNA measurements of 134 surgically obtained OSCCs and 30 control samples to analyze the expression of the relevant target mRNAs (Table 1) 19.

**Table 1:** Neurotrophin signaling pathway targeted genes and expression level in Oral squamous cell carcinoma and normal tissue in TCGA samples. FDR is false discovery rate

Gene	Fold Change	Tumor Level	Nontumor Level	FDR
CAMK2D	No Change			
BRAF	No Change			
GSK3B	1.36	1929	1416	0.00082
**NTRK2**	0.29	416	1460	0.01
NFKB1	No Change			
SOS2	NA			
RAPGEF1	No Change			
SH2B3	1.67	656	392	0.0016
NRAS	1.55	2175	1400	0.00001
CRKL	1.62	3560	2196	0.0000024
PIK3CB	No Change			
MAPK7	No Change			
MAP2K7	No Change			
**SORT1**	0.37	1422	3810	0.0000001
PIK3R2	1.07			0.45
**BCL2**	0.42	131	311	0.000013
MAP3K1	0.69	747	1076	0.0044
NGFRAP1	1.63	1564	958	0.000418
MAGED1	No Change			
ARHGDIB	No Change			
FASLG	No Change			
MAPK13	0.76	1579	2075	0.173
RPS6KA5	0.62	176	286	0.0005
TP53	0.69	966	1406	0.102
PLCG1	1.64	1409	862	0.0000001
**PIK3CD**	2.65	859	323	0.0000001
JUN	0.81	6250	7702	0.193
PIK3R3	0.59	385	650	0.0022
TRAF6	No Change			
AKT1	No Change			
**RPS6KA6**	0.066	1.98	30	0.0000001
**PIK3R1**	0.42	851	2024	0.0000002
IRS1	1.86	1487	800	0.000447
CDC42	No Change			
PLCG2	1.3	407	313	0.0922
GAB1	0.69	595	868	0.00469
AKT3	1.52	292	192	0.034
PIK3CA	1.52	642	423	0.000438
FOXO3	No Change			
SH2B1	0.69	617	891	0.00033
IRAK1	1.57	4523	2883	0.000008
ABL1	No Change			
ATF4	0.75	4968	6651	0.0015
MAP3K5	1.18	786	667	0.197

## Discussion

Much work has gone into detection and diagnosis of OSCC based on RNA changes with the disease. The large set of oral cancer samples included in the TCGA dataset, harvested under standardized conditions by surgery, facilitates an analysis of detection of this disease as the number of samples is high enough to allow extensive statistical analysis. In addition, several studies demonstrated the heterogeneity of gene expression and miRNA expression in head and neck and OSCC cancers, suggesting that the development of an RNA‐based classifier for OSCC may be complex 19, 20, 21, 35. Despite that, we were able to use the TCGA dataset to show a robust classifier for OSCC based on miRNA expression can be developed with as little as 10 OSCC and 10 controls. Given this positive result with surgical samples, we tested brush biopsy samples from cancer and healthy tissue and saw accuracy of OSCC identification at 87% (Fig. 3). We also saw 37% agreement in expression profiles between those generated from brush biopsy versus the surgically obtained samples for the TCGA study (supplemental data). That it is not higher may in part be explained by the fact that TCGA tumor samples can be as much as 50% stroma and the controls as high as 100% stroma 19. Comparisons of the brush biopsy miRNA list to a second list of OSCC‐associated miRNA from surgical OSCC samples of Lager et al. 5 showed only slightly more agreement at 43%. We speculate that the list of differentially expressed miRNA in brush biopsy samples would not show changes that occur in stroma. The brush biopsy samples are instead more sensitive to changes in epithelium. For example, if a miRNA changes from 50 copies per cells to 5000 with OSCC, whereas the level in stroma is a constant 10,000 copies per cell, this difference will easily be seen in brush biopsy samples but not in surgical samples that contain variable amounts of stroma. For that reason we do not expect 100% agreement with the miRNA list generated by brush biopsy of OSCC versus healthy mucosa compared to that seen with surgically obtained samples even under ideal conditions.

It is not clear why some tumor samples showed variable identification as tumor depending on the algorithm used. The three tumors that were difficult to classify, of 21 total, included two early, classified as T1N0M0, and one late, T3N0M0. All three were reconfirmed as correctly diagnosed by a third oral pathologist's review of the stained tissue. One possible explanation is unusual miRNA expression present in these tumors, suggesting more training of the classifier is needed. Another source of error is that the site of OSCC was not sampled. This can occur in large lesions where much of the superficial oral epithelium is changed in appearance but only a small site is transformed. This is also one cause of tumor misdiagnosis by histopathology, which is estimated to happen over 10% of the time after the initial surgical biopsy, the gold standard 38. Use of multiple sites of brush biopsy in large lesions would assist with pinpointing the malignant site, which would aid in surgical treatment.

Saliva studies have identified miRNAs differentially expressed with OSCC 17, 18, 36, 39. Studies have examined miRNA from whole saliva, which would include shed cells, and saliva supernatant, which may contain secreted RNA and RNA from lysed cells. We saw some overlap between our study and these saliva studies of OSCC‐associated miRNAs. Two miRNAs of five identified by Salazar et. al., miR‐127‐3p and miR‐9‐5p, were also shown to change in our brush biopsy study 15. Likewise, one of four differentially expressed miRNAs in saliva of OSCC patients, miR‐142‐3p, was identified in this work 14. There was also overlap between miRNA markers identified in our brush biopsy study and those of saliva supernatant, one of three in one study, miR‐21 54 and miR‐21 and miR‐31 elevated in a saliva supernatant study that focused on these two miRNAs 10, though at best only 1 of 13 in another, miR‐147 13. The similarity of miRNA differentially expressed in most cases, whether we compared studies done with whole saliva or cell‐free saliva to RNA from brush biopsy, suggests saliva contains a large portion of miRNA nonspecifically released from tumor cells and not specifically secreted 16. Surprisingly, an examination of miRNAs in blood fluids of OSCC patients in one study showed at least five of 12 miRNAs differentially expressed in serum were also differentially expressed in OSCC epithelium in our study, miR‐7, miR‐26a, miR‐17, miR‐19a‐3p, miR‐486‐5p, and possibly miR‐16 and miR‐30e 40. This study used the same platform to examine miRNA levels as used in this study, but others that did not also showed similar changes of levels in plasma for miR‐31, miR‐10b, and miR‐146a of OSCC patients and our brush biopsy obtained cells 10, 11, 12, 17. Though miRNA fold changes in blood fluids tend to be dampened compared to those found in tumor epithelium probably due to the presence of miRNA from other sources. Like saliva, the correspondence in the identification of OSCC miRNA markers in blood and our brush biopsy samples of tumor epithelium suggest the source is nonspecific release of miRNA from tumor cells.

This study serves as proof of principle that it is possible to develop a miRNA signature to differentiate OSCC from nondiseased healthy tissue using brush biopsy. We saw strong correlations between the two platforms, with seven of nine miRNA differentially expressed by miRNAseq and tested in both studies also differentially expressed when measured by RT‐PCR at *P* < 0.05. Also presented is a list of old and newly identified miRNAs in epithelium that can be used to differentiate OSCC from healthy tissue. Of course, to detect cancer what is needed is a classifier that can differentiate OSCC from benign disease. Any attempt to develop a classifier to identify OSCC in the clinic will require samples from multiple subjects with conditions sometime mistaken for OSCC such as lichen planus, leukoplakia, and submucosal fibrosis to train the classifier to distinguish these lesions from OSCC based on the described and additional miRNAs. Thus brush biopsy has the potential to deliver a diagnostic for oral mucosal disease that will potentially allow early detection and diagnosis of all these disease types without surgical incisional biopsy and without referral.

The epithelium‐specific miRNA analysis revealed one pathway linked to OSCC based on changes in miRNA levels identified in this work and after elimination of pathways that appeared in the negative control analyses (Data S1). This was the neurotrophin signaling pathway (Fig. 4). It can regulate cell survival, angiogenesis, tissue invasion, DNA damage resistance, and EMT 41, 42, 43, 44, 45. Of the six mRNAS identified in the neurotrophin signaling pathway targeted by the miRNAs (Table 1) that showed at least 2× fold change in level, RPS6KA6 and PIK3R1 tumor suppressors that work to inhibit the oncogene PIK3CA were both decreased 46, 47, 48. SORT1 mRNA was also decreased. A decrease in SORT1 may play a role in the increase in the low‐affinity nerve growth receptor p75TNR that is often seen with OSCC 49, 50. The oncogene BCL2 mRNA counterintuitively was decreased, as was the high‐affinity nerve growth receptor mRNA, NTRK2. There is fairly good evidence NTRK2, one of several neurotrophin receptors, is enriched on the RNA and protein level in pharyngeal cancer and there is limited evidence for this in OSCC 43, 44, 51. Our results and that of the TCGA data analysis instead are more compatible with a decrease. We note in various squamous cell type carcinomas that NTRK2 has mixed properties; it has shown expression patterns consistent with the inhibition of tumor progression in SCC lung cancer and cervical cancer, whereas in cutaneous and basal SCC, NTRK2 is enriched at sites of nerve invasion, and in vitro it can promote mesenchymal epithelial transition and chemotherapy resistance in head and neck cancer cell lines 41, 43, 52, 53. This work shows the importance of the neurotrophin signaling pathway in OSCC formation and progression, but much remains to be known about its role in the process or if it regulates the neural invasion that occurs in aggressive OSCC 50.

## Conclusions

Brush biopsy offers a method to identify cancer specific to the tumor, as samples come directly from suspicious lesions, and it aids in tumor localization. An accurate saliva‐based method would offer the easiest method to obtain samples to screen for OSCC. While many sources of error in saliva miRNA analysis can be eliminated by optimization, the most troubling will be errors introduced due to recent food intake or usage of oral hygiene products that are already known to have a large effect on saliva content. Serum, which contains RNA markers from a variety of malignancies, showed a remarkable correspondence to miRNA changes observed from brush oral biopsy of tumor lesions despite acquisition variables. In regions of the world where OSCC has very high incidence rates, any of these approaches to OSCC detection would be especially helpful as the incidence rate is high enough that even conventional OSCC screening has shown positive effects on patient outcomes 28. Finally, an adaptation of a new method to identify molecular pathways targeted by miRNAs 34 was used to show regulation of the neurotrophin signaling pathway, which may play an important role in OSCC formation and progression.

## Conflict of Interest

None declared.

## Supporting information


**Figure S1.** Levels of miR‐503‐5p and miR‐196a‐5p in vivo and in vitro in epithelium differ.

**Table S1.** Clinical characterization of the subject groups.
**Table S2.** Clinical Information for OSCC subjects specific to this study.
**Table S3.** RNAs that make up TCGA‐based OSCC class predictor 2.
**Table S4.** RNAs that make up TCGA‐based OSCC class predictor 2.
**Table S5.** RNAs that makeup TCGA‐based OSCC Class Predictor 3.
**Table S6** RNAs that make up Brush Cytology based class predictor for OSCC with miRNAseq quantitation.
**Table S7.** RNAs that make up Brush Cytology based class predictor for OSCC with qRT‐PCR‐based quantitation.
**Table S8.** Pathways targeted by OSCC specific 11 miRNAs (hsa‐miR‐486‐5p, hsa‐miR‐7‐5p, hsa‐miR‐146b‐5p, hsa‐miR‐101‐3p, hsa‐miR‐18b‐5p, hsa‐miR‐10b‐5p, hsa‐miR‐21‐5p, hsa‐miR‐190a‐5p, hsa‐miR‐20b‐5p, hsa‐miR‐126‐3p, and hsa‐miR 31‐5p) as identified by mirPath v.3.
**Table S9.** Pathways targeted by OSCC specific 11 miRNAs (hsa‐miR‐486‐5p, hsa‐miR‐7‐5p, hsa‐miR‐146b‐5p, hsa‐miR‐101‐3p, hsa‐miR‐18b‐5p, hsa‐miR‐10b‐5p, hsa‐miR‐21‐5p, hsa‐miR‐190a‐5p, hsa‐miR‐20b‐5p, hsa‐miR‐126‐3p, and hsa‐miR 31‐5p) as identified by mirPath v.3 that overlap with those predicted to be targeted by the 20 sets of 11 random control miRNAs.

**Data S1.** Human OSCC cell lines, HSC‐2, HSC‐3, HSC‐4, and Ca9‐22 cells, were obtained from the Japanese Collection of Research Bioresources Cell Bank, National Institute of Biomedical Innovation.
