# Supplementary figures and images for: microRNA from brush biopsy to characterize oral squamous cell carcinoma epithelium
Source: Cancer Med. 2016 Dec 18;6(1):67–78. doi: 10.1002/cam4.951 (PMC5275769; doi:10.1002/cam4.951)

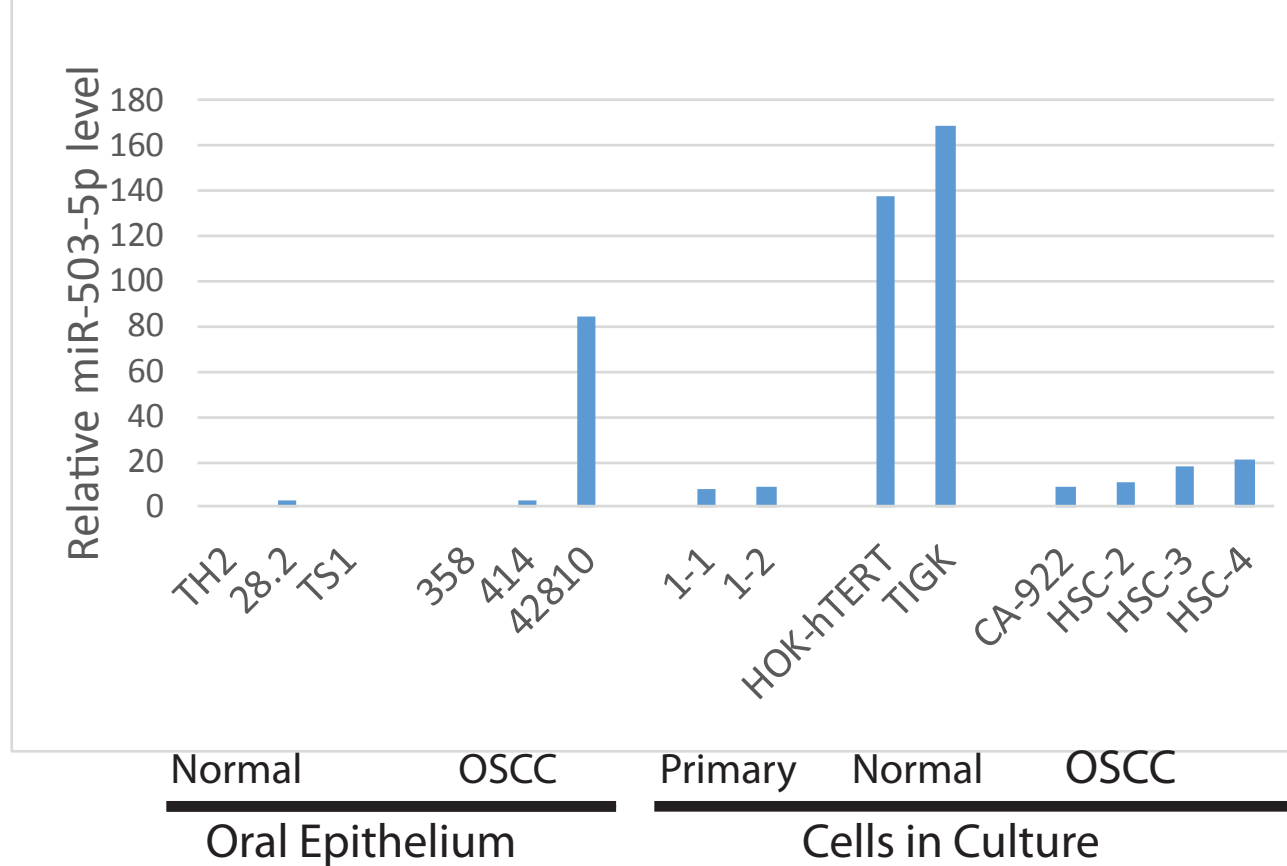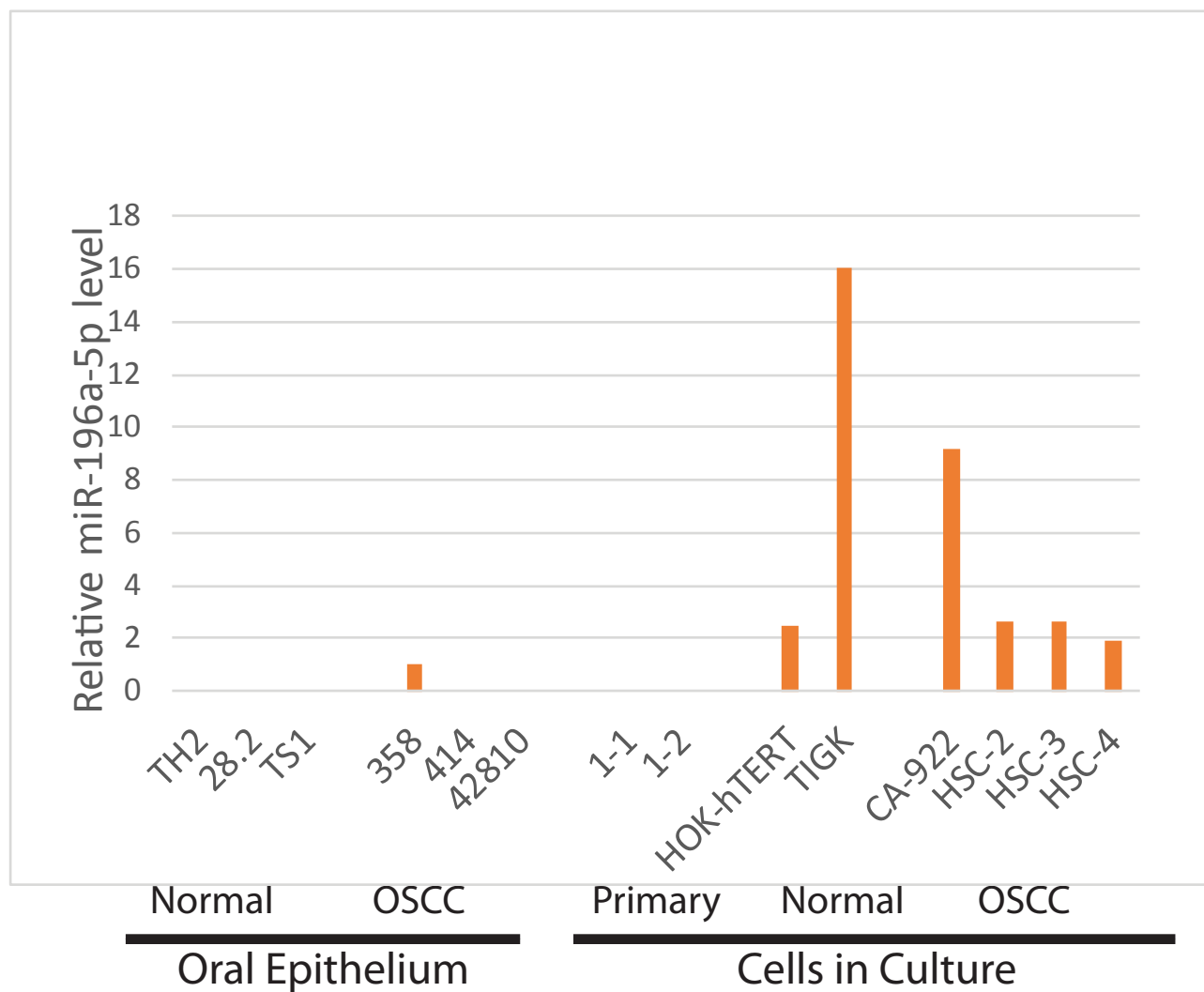

Supplement: Supplementary file 1 — Figure S1. Levels of miR‐503‐5p and miR‐196a‐5p in vivo and in vitro in epithelium differ. [file CAM4-6-67-s001.pdf]
