# Supplementary material for: microRNA from brush biopsy to characterize oral squamous cell carcinoma epithelium
Source: Cancer Med. 2016 Dec 18;6(1):67–78. doi: 10.1002/cam4.951 (PMC5275769; doi:10.1002/cam4.951)

| Supplemental Table 1 Clinical characterization of the subject groups |  |  |  |  |
| --- | --- | --- | --- | --- |

| Method of RNA analysis | miRNAseq |  |  | RT-PCR |  |
| --- | --- | --- | --- | --- | --- |
| Status | OSCC | Normal |  | OSCC | Normal |
| Total Number of Subjects | 20 | 7 |  | 20 | 17 |
| Age | 37-90, 61.5 | 26-71, 56 |  | 37-90, 62 | 26-76, 52 |
|  |  |  |  |  |  |
| Gender | 12M/8F | 3M/4F |  | 12M/8F | 11M/7F |
|  |  |  |  |  |  |
| Site^b^ | 10 T, 7 LG, 2 FOM, 1BU | 4T, 3LM |  | 10T, 8LG, 1Bu, 1FOM | 13T, 3LG, 1 Bu |
|  |  |  |  |  |  |
| History of Tobacco/Betel Nut | 9 | 0 |  | 8 | 8 |
|  |  |  |  |  |  |
| Tumor Size |  |  |  |  |  |
| T1/T2 | 11 |  |  | 10 |  |
| T3/T4 | 9^c^ |  |  | 10^d^ |  |

| a. Tumor samples were acquired earlier or for this study(see table 2) |
| --- |
| b. Tongue, T; Lower Gingiva, LG; Floor of Mouth, FOM; Buccal, Bu |
| c. two T4N2M0 |
| d. two T4N2Mo |

Supplemental Table 2 Clinical Information for OSCC subjects specific to this study

|  | Site | Gender | Age | History of Exposure | Classification | Grade |
| --- | --- | --- | --- | --- | --- | --- |
| OSCC383 | T | M | 45 | Betel | T4AlphaN0M0 | II |
| OSCC 578 | T | F | 57 | Tobacco | T1N0M0 | I |
| OSCC583 | T | M | 56 | Tobacco | T1N0M0 | I |
| OSCC589 | FOM | M | 69 | Tobacco | T1N0M0 | II |

| a. Tongue, T; Floor of Mouth, FOM |  |  |  |  |
| --- | --- | --- | --- | --- |

Supplemental Table 3 RNAs that make up TCGA based OSCC class predictor 2

|  | 95% Parametric p-value | Fold-change | UniqueID |
| --- | --- | --- | --- |
| 1 | < 1e-07 | 0.036 | hsa-mir-204 |
| 2 | < 1e-07 | 0.24 | hsa-mir-101-1 |
| 3 | < 1e-07 | 6.25 | hsa-mir-550a-1 |
| 4 | 0.0000009 | 0.13 | hsa-mir-29c |
| 5 | 0.0000011 | 0.11 | hsa-let-7c |
| 6 | 0.0000012 | 6.08 | hsa-mir-550a-2 |
| 7 | 0.0000014 | 4.94 | hsa-mir-424 |
| 8 | 0.0000035 | 0.073 | hsa-mir-99a |
| 9 | 0.0000042 | 4.18 | hsa-mir-450b |
| 10 | 0.0000044 | 11 | hsa-mir-503 |
| 11 | 0.0000063 | 7.8 | hsa-mir-455 |
| 12 | 0.0000063 | 2.73 | hsa-mir-324 |
| 13 | 0.0000066 | 0.24 | hsa-mir-139 |
| 14 | 0.0000077 | 21.73 | hsa-mir-31 |
| 15 | 0.0000098 | 4.12 | hsa-mir-16-2 |
| 16 | 0.0000164 | 0.084 | hsa-mir-125b-2 |
| 17 | 0.0000286 | 0.18 | hsa-mir-30a |
| 18 | 0.000029 | 0.47 | hsa-mir-140 |
| 19 | 0.0000308 | 2.71 | hsa-mir-15b |
| 20 | 0.0000337 | 0.34 | hsa-mir-29a |
| 21 | 0.0000419 | 4.9 | hsa-mir-1292 |
| 22 | 0.0000439 | 5.31 | hsa-mir-877 |
| 23 | 0.0000536 | 14.29 | hsa-mir-196b |
| 24 | 0.0000539 | 3.46 | hsa-mir-183 |
| 25 | 0.0000942 | 7.12 | hsa-mir-224 |
| 26 | 0.0000947 | 3.03 | hsa-mir-454 |
| 27 | 0.0001096 | 0.17 | hsa-mir-410 |
| 28 | 0.0001271 | 3.67 | hsa-mir-21 |
| 29 | 0.0001313 | 3.11 | hsa-mir-1301 |
| 30 | 0.0001575 | 6.03 | hsa-mir-1245 |
| 31 | 0.0001767 | 0.19 | hsa-mir-100 |
| 32 | 0.0001779 | 6 | hsa-mir-301a |
| 33 | 0.0001816 | 13.23 | hsa-mir-196a-1 |
| 34 | 0.0001817 | 8.81 | hsa-mir-3648 |
| 35 | 0.0002233 | 3.5 | hsa-mir-193b |
| 36 | 0.0002382 | 2.29 | hsa-mir-576 |
| 37 | 0.0002394 | 0.47 | hsa-mir-30e |
| 38 | 0.0002407 | 2.95 | hsa-mir-484 |
| 39 | 0.0002538 | 3.4 | hsa-mir-3074 |
| 40 | 0.0002541 | 4.1 | hsa-mir-3928 |
| 41 | 0.0002654 | 0.037 | hsa-mir-375 |
| 42 | 0.000281 | 0.25 | hsa-mir-195 |
| 43 | 0.0002919 | 3.8 | hsa-mir-450a-2 |
| 44 | 0.0003267 | 0.29 | hsa-mir-125b-1 |
| 45 | 0.0004122 | 2.26 | hsa-mir-1306 |
| 46 | 0.000435 | 3.28 | hsa-mir-450a-1 |
| 47 | 0.0004397 | 2.63 | hsa-mir-96 |
| 48 | 0.0004456 | 11.05 | hsa-mir-937 |
| 49 | 0.000449 | 7.71 | hsa-mir-615 |
| 50 | 0.0004689 | 4.12 | hsa-mir-2355 |

Supplemental Table 4 RNAs that make up TCGA based OSCC class predictor 2

|  | 90%Parametric p-value | Fold-change | UniqueID |
| --- | --- | --- | --- |
| 1 | < 1e-07 | 0.22 | hsa-mir-101-1 |
| 2 | 0.0000013 | 0.098 | hsa-mir-125b-2 |
| 3 | 0.0000018 | 0.091 | hsa-mir-99a |
| 4 | 0.0000028 | 7.15 | hsa-mir-4326 |
| 5 | 0.0000033 | 0.11 | hsa-let-7c |
| 6 | 0.0000185 | 2.68 | hsa-mir-130b |
| 7 | 0.0000201 | 2.07 | hsa-mir-423 |
| 8 | 0.0000358 | 36.4 | hsa-mir-196a-1 |
| 9 | 0.0000433 | 0.51 | hsa-mir-30e |
| 10 | 0.0000604 | 2.38 | hsa-mir-671 |
| 11 | 0.0001043 | 3.84 | hsa-mir-1301 |
| 12 | 0.0001127 | 10.78 | hsa-mir-196b |
| 13 | 0.0001289 | 2.08 | hsa-mir-501 |
| 14 | 0.0002065 | 4.63 | hsa-mir-3662 |
| 15 | 0.000234 | 9.48 | hsa-mir-1293 |
| 16 | 0.0003316 | 2.25 | hsa-mir-197 |
| 17 | 0.0004565 | 0.33 | hsa-mir-100 |

Supplemental Table 5 RNAs that makeup TCGA based OSCC Class Predictor 3

|  | 100%Parametric p-value | Fold-change | UniqueID |
| --- | --- | --- | --- |
| 1 | 0.000001 | 0.22 | hsa-mir-101-2 |
| 2 | 0.0000032 | 0.26 | hsa-mir-101-1 |
| 3 | 0.0000074 | 0.081 | hsa-mir-204 |
| 4 | 0.0000137 | 0.11 | hsa-mir-891a |
| 5 | 0.0000084 | 0.4 | hsa-mir-140 |
| 6 | 0.0000138 | 0.19 | hsa-mir-99a |
| 7 | 0.0000216 | 0.25 | hsa-mir-1468 |
| 8 | 0.0000388 | 0.17 | hsa-mir-410 |
| 9 | 0.0000446 | 0.18 | hsa-mir-30a |
| 10 | 0.0000482 | 0.26 | hsa-mir-432 |
| 11 | 0.0000491 | 0.23 | hsa-mir-29c |
| 12 | 0.0000645 | 0.036 | hsa-mir-375 |
| 13 | 0.0001122 | 0.35 | hsa-mir-195 |
| 14 | 0.0001866 | 0.29 | hsa-mir-487b |
| 15 | 0.0002036 | 0.35 | hsa-mir-100 |
| 16 | 0.000212 | 0.23 | hsa-mir-125b-2 |
| 17 | 0.0002185 | 0.23 | hsa-mir-376c |
| 18 | 0.0003111 | 0.35 | hsa-mir-656 |
| 19 | 0.0002901 | 0.45 | hsa-mir-125b-1 |
| 20 | 0.0003015 | 0.25 | hsa-let-7c |
| 21 | 0.0003401 | 0.13 | hsa-mir-381 |
| 22 | 0.0003673 | 0.37 | hsa-mir-889 |
| 23 | 0.0003979 | 0.28 | hsa-mir-431 |
| 24 | 0.0004061 | 0.29 | hsa-mir-369 |
| 25 | 0.0004301 | 0.19 | hsa-mir-299 |
| 26 | 0.0004378 | 0.44 | hsa-mir-30e |
| 27 | 0.0004526 | 0.26 | hsa-mir-217 |
| 28 | 0.0004923 | 2.52 | hsa-mir-421 |
| 29 | 0.0004873 | 4.17 | hsa-mir-3677 |
| 30 | 0.0004682 | 2.54 | hsa-mir-584 |
| 31 | 0.0004323 | 2.89 | hsa-mir-550a-2 |
| 32 | 0.0004002 | 5.17 | hsa-mir-944 |
| 33 | 0.0003761 | 2.43 | hsa-mir-181b-1 |
| 34 | 0.0003667 | 3.34 | hsa-mir-183 |
| 35 | 0.000346 | 2.21 | hsa-mir-15b |
| 36 | 0.0003771 | 3.33 | hsa-mir-940 |
| 37 | 0.0003717 | 2.9 | hsa-mir-939 |
| 38 | 0.0003159 | 2.49 | hsa-mir-505 |
| 39 | 0.0002991 | 1.69 | hsa-mir-652 |
| 40 | 0.0003796 | 4.79 | hsa-mir-3928 |
| 41 | 0.0002877 | 3.79 | hsa-mir-592 |
| 42 | 0.0002729 | 3.41 | hsa-mir-550a-1 |
| 43 | 0.000253 | 2.79 | hsa-mir-92b |
| 44 | 0.0002139 | 2.33 | hsa-mir-330 |
| 45 | 0.0002045 | 3.19 | hsa-mir-222 |
| 46 | 0.0001767 | 1.92 | hsa-mir-148b |
| 47 | 0.0002633 | 3.27 | hsa-mir-3922 |
| 48 | 0.0001621 | 3.9 | hsa-mir-21 |
| 49 | 0.0001471 | 1.87 | hsa-mir-106b |
| 50 | 0.0001243 | 2.93 | hsa-mir-1301 |
| 51 | 0.000116 | 3.74 | hsa-mir-3934 |
| 52 | 0.0000935 | 4.31 | hsa-mir-450a-2 |
| 53 | 0.0000703 | 2.08 | hsa-let-7d |
| 54 | 0.0000681 | 6.3 | hsa-mir-301a |
| 55 | 0.0000785 | 2.58 | hsa-mir-3074 |
| 56 | 0.0000508 | 3.22 | hsa-mir-1307 |
| 57 | 0.000041 | 2.68 | hsa-mir-450b |
| 58 | 0.000025 | 4 | hsa-mir-3605 |
| 59 | 0.0000112 | 4.12 | hsa-mir-2355 |
| 60 | 0.000011 | 2.91 | hsa-mir-766 |
| 61 | 0.0000098 | 2.72 | hsa-mir-744 |
| 62 | 0.0000087 | 3.17 | hsa-mir-331 |
| 63 | 0.000006 | 3.61 | hsa-mir-345 |
| 64 | 0.0000052 | 2.38 | hsa-mir-7-1 |
| 65 | 0.0000039 | 3.29 | hsa-mir-130b |
| 66 | 0.0000035 | 11.34 | hsa-mir-877 |
| 67 | 0.0000019 | 2.63 | hsa-mir-671 |
| 68 | 0.0000016 | 38.08 | hsa-mir-196a-1 |
| 69 | 0.0000008 | 12.77 | hsa-mir-503 |
| 70 | 0.000001 | 9.27 | hsa-mir-937 |
| 71 | 0.0000063 | 7.94 | hsa-mir-1910 |
| 72 | 0.0000005 | 4.66 | hsa-mir-193b |
| 73 | 0.0000004 | 3.86 | hsa-mir-324 |
| 74 | 0.0000004 | 40.46 | hsa-mir-196b |
| 75 | 0.0000232 | 24.39 | hsa-mir-615 |
| 76 | 0.0000002 | 7.7 | hsa-mir-187 |
| 77 | 0.0000002 | 2.87 | hsa-mir-1306 |
| 78 | 0.0000002 | 6.21 | hsa-mir-424 |
| 79 | 0.0000002 | 13.81 | hsa-mir-3940 |
| 80 | < 1e-07 | 10.39 | hsa-mir-455 |

Supplemental Table 6 RNAs that make up Brush Cytology based class predictor for OSCC with miRNAseq quantitation

|  | Parametric p-value | Fold-change | UniqueID |
| --- | --- | --- | --- |
| 1 | 0.0002033 | 4 | hsa-miR-3605-3p |
| 2 | 0.0002462 | 11.22 | hsa-miR-10a-5p |
| 3 | 0.000332 | 13.07 | hsa-miR-10b-5p |
| 4 | 0.0003518 | 5.08 | hsa-miR-185-3p |
| 5 | 0.0011606 | 4.38 | hsa-miR-424-5p |
| 6 | 0.0013125 | 4.8 | hsa-miR-99b-3p |
| 7 | 0.0016351 | 1.89 | hsa-miR-339-5p |
| 8 | 0.0022419 | 2.42 | hsa-miR-328-3p |
| 9 | 0.0029416 | 5.33 | hsa-miR-126-5p |
| 10 | 0.0034308 | 2.71 | hsa-miR-31-3p |
| 11 | 0.004026 | 0.57 | hsa-miR-200b-5p |
| 12 | 0.0041133 | 21.09 | hsa-miR-196a-5p |
| 13 | 0.0059159 | 9.12 | hsa-miR-190a-5p |
| 14 | 0.0079018 | 2.11 | hsa-miR-31-5p |
| 15 | 0.0086229 | 3.44 | hsa-miR-766-3p |

Supplemental Table 7 RNAs that make up Brush Cytology based class predictor for OSCC with qRT-PCR based quantitation

|  | Parametric p-value | Fold-change | UniqueID |
| --- | --- | --- | --- |
| 1 | 0.0000096 | 47.03 | hsa-miR-486-5p |
| 2 | 0.0000407 | 6 | hsa-mir-7-5p |
| 3 | 0.0000535 | 2.59 | hsa-miR-146b-5p |
| 4 | 0.0000667 | 0.51 | hsa-miR-130b-3p |
| 5 | 0.0000683 | 2.65 | hsa-miR-101-3p |
| 6 | 0.0000869 | 2.02 | hsa-miR-18b-5p |
| 7 | 0.0001101 | 43.97 | hsa-miR-10b-5p |
| 8 | 0.0001448 | 2.65 | hsa-miR-21-5p |
| 9 | 0.0001769 | 8.23 | hsa-miR-190a |
| 10 | 0.000233 | 5.55 | hsa-miR-20b-5p |
| 11 | 0.0002736 | 7.39 | hsa-miR-126-3p |
| 12 | 0.0002888 | 4.66 | hsa-miR-31-5p |
| 13 | 0.0003458 | 0.48 | hsa-miR-34a-5p |
| 14 | 0.0004278 | 3.5 | hsa-miR-100-5p |
| 15 | 0.0004544 | 1.95 | hsa-miR-19a-3p |
| 16 | 0.0005441 | 8.3 | hsa-miR-199a-5p |
| 17 | 0.000667 | 0.32 | hsa-miR-296-5p |
| 18 | 0.0006819 | 1.84 | hsa-miR-18a-5p |
| 19 | 0.0006857 | 0.18 | hsa-miR-885-5p |
| 20 | 0.0007666 | 0.61 | hsa-miR-378a-3p |
| 21 | 0.0008715 | 0.49 | hsa-miR-210 |
| 22 | 0.0009588 | 0.59 | hsa-miR-324-3p |
| 23 | 0.0009687 | 0.16 | hsa-miR-30b-3p |
| 24 | 0.001268 | 6.85 | hsa-miR-127-3p |
| 25 | 0.0012812 | 0.61 | hsa-miR-365a-3p |
| 26 | 0.0012911 | 1.98 | hsa-miR-194-5p |
| 27 | 0.0014138 | 3.11 | hsa-miR-671-5p |
| 28 | 0.0016244 | 0.042 | hsa-miR-340-5p |
| 29 | 0.0016916 | 0.51 | hsa-miR-423-5p |
| 30 | 0.0017902 | 0.3 | hsa-miR-375 |
| 31 | 0.0017916 | 3.46 | hsa-miR-155-5p |
| 32 | 0.0020139 | 7.19 | hsa-miR-187-3p |
| 33 | 0.0021023 | 1.52 | hsa-miR-17-5p |
| 34 | 0.0022965 | 2.46 | hsa-miR-454-3p |
| 35 | 0.0025843 | 2.96 | hsa-miR-363-3p |
| 36 | 0.0030432 | 1.48 | hsa-miR-106a-5p |
| 37 | 0.0033991 | 0.35 | hsa-miR-218-5p |
| 38 | 0.0034229 | 2.44 | hsa-miR-135b-5p |
| 39 | 0.0044533 | 1.61 | hsa-miR-19b-3p |
| 40 | 0.0044576 | 2.64 | hsa-miR-135a-5p |
| 41 | 0.0045035 | 3.25 | hsa-miR-146a-5p |
| 42 | 0.0047201 | 0.17 | hsa-miR-345-5p |
| 43 | 0.0047608 | 0.59 | hsa-miR-574-3p |
|  |  |  |  |

Supplemental Table 8 Pathways targeted by OSCC specific 11 miRNAs (hsa-miR-486-5p, hsa-miR-7-5p, hsa-miR-146b-5p, hsa-miR-101-3p, hsa-miR-18b-5p, hsa-miR-10b-5p, hsa-miR-21-5p, hsa-miR-190a-5p, hsa-miR-20b-5p, hsa-miR-126-3p, and hsa-miR 31-5p) as identified by mirPath v.3.

| **KEGG pathway** | **p-value** | **#genes** | **#miRNAs** |
| --- | --- | --- | --- |
| MicroRNAs in cancer | 5.07E-65 | 105 | 10 |
| Proteoglycans in cancer | 2.55E-13 | 104 | 10 |
| Hepatitis B | 2.12E-12 | 81 | 10 |
| Fatty acid biosynthesis | 1.98E-11 | 6 | 6 |
| Pancreatic cancer | 1.98E-11 | 48 | 10 |
| Colorectal cancer | 3.88E-11 | 46 | 10 |
| Non-small cell lung cancer | 6.15E-11 | 41 | 10 |
| Glioma | 2.08E-10 | 42 | 10 |
| Chronic myeloid leukemia | 2.33E-10 | 51 | 10 |
| Viral carcinogenesis | 4.10E-10 | 96 | 10 |
| Neurotrophin signaling pathway | 5.36E-09 | 74 | 10 |
| Prion diseases | 1.32E-08 | 14 | 8 |
| Renal cell carcinoma | 5.65E-08 | 45 | 10 |
| Endometrial cancer | 5.82E-08 | 37 | 10 |
| Pathways in cancer | 9.03E-08 | 181 | 10 |
| Thyroid hormone signaling pathway | 2.21E-07 | 64 | 10 |
| FoxO signaling pathway | 2.71E-07 | 77 | 10 |
| Cell cycle | 7.30E-07 | 70 | 10 |
| Prostate cancer | 7.70E-07 | 56 | 10 |
| Lysine degradation | 2.18E-06 | 28 | 9 |
| Bladder cancer | 2.18E-06 | 29 | 10 |
| TGF-beta signaling pathway | 6.75E-06 | 39 | 10 |
| Protein processing in endoplasmic reticulum | 9.85E-06 | 86 | 10 |
| Adherens junction | 9.85E-06 | 42 | 10 |

Supplemental Table 9 Pathways targeted by OSCC specific 11 miRNAs (hsa-miR-486-5p, hsa-miR-7-5p, hsa-miR-146b-5p, hsa-miR-101-3p, hsa-miR-18b-5p, hsa-miR-10b-5p, hsa-miR-21-5p, hsa-miR-190a-5p, hsa-miR-20b-5p, hsa-miR-126-3p, and hsa-miR 31-5p) as identified by mirPath v.3 that overlap with those predicted to be targeted by the 20 sets of 11 random control miRNAs.


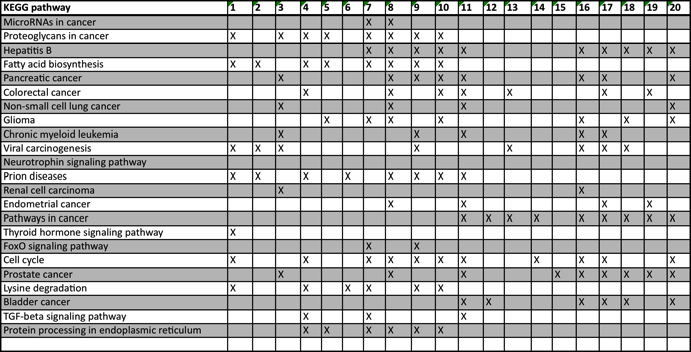

Supplement: Supplementary file 2 — Table S1. Clinical characterization of the subject groups. Table S2. Clinical Information for OSCC subjects specific to this study. Table S3. RNAs that make up TCGA‐based OSCC class predictor 2. Table S4. RNAs that make up TCGA‐based OSCC class predictor 2. Table S5. RNAs that makeup TCGA‐based OSCC Class Predictor 3. Table S6 RNAs that make up Brush Cytology based class predictor for OSCC with miRNAseq quantitation. Table S7. RNAs that make up Brush Cytology based class predictor for OSCC with qRT‐PCR‐based quantitation. Table S8. Pathways targeted by OSCC specific 11 miRNAs (hsa‐miR‐486‐5p, hsa‐miR‐7‐5p, hsa‐miR‐146b‐5p, hsa‐miR‐101‐3p, hsa‐miR‐18b‐5p, hsa‐miR‐10b‐5p, hsa‐miR‐21‐5p, hsa‐miR‐190a‐5p, hsa‐miR‐20b‐5p, hsa‐miR‐126‐3p, and hsa‐miR 31‐5p) as identified by mirPath v.3. Table S9. Pathways targeted by OSCC specific 11 miRNAs (hsa‐miR‐486‐5p, hsa‐miR‐7‐5p, hsa‐miR‐146b‐5p, hsa‐miR‐101‐3p, hsa‐miR‐18b‐5p, hsa‐miR‐10b‐5p, hsa‐miR‐21‐5p, hsa‐miR‐190a‐5p, hsa‐miR‐20b‐5p, hsa‐miR‐126‐3p, and hsa‐miR 31‐5p) as identified by mirPath v.3 that overlap with those predicted to be targeted by the 20 sets of 11 random control miRNAs. [file CAM4-6-67-s002.docx]
