# Supplementary material for: microRNA from brush biopsy to characterize oral squamous cell carcinoma epithelium
Source: Cancer Med. 2016 Dec 18;6(1):67–78. doi: 10.1002/cam4.951 (PMC5275769; doi:10.1002/cam4.951)
Supplement: Supplementary file 3 — Data S1. Human OSCC cell lines, HSC‐2, HSC‐3, HSC‐4, and Ca9‐22 cells, were obtained from the Japanese Collection of Research Bioresources Cell Bank, National Institute of Biomedical Innovation. [file CAM4-6-67-s003.doc]

Supplemental Methods

Human OSCC cell lines, HSC-2, HSC-3, HSC-4 and Ca9-22 cells, were obtained from the Japanese Collection of Research Bioresources Cell Bank, National Institute of Biomedical Innovation. The human OSCC cell line, SCC-9 cells, was obtained from the American Type Culture Collection. These cells were cultured in Dulbecco’s Modified Eagle Medium (Gibco, Grand Island, NY, USA) with 10% fetal bovine serum (Intergen) and 100 U/mL of penicillin sodium (Invitrogen, Life Technologies, California, USA) and 100 mg/mL of streptomycin sulfate (Invitrogen, Life Technologies). During some expression assays Keratinocyte SFM serum free medium described below was used instead 48 hours prior to RNA harvest. This had no effect on measured RNA levels. All cells were incubated at 37°C in 5% CO2 atmosphere. Primary cell strains were obtained and prepared from discarded gingival epithelium from extracted molars as described earlier and cultured in Keratinocyte-SFM serum free medium (Gibco, Grand Island, NY, USA) supplemented with 200 mM CaCl2. The TIGK line was obtained from Dr. Richard Lamont with the help of Dr. Louisa Di Pietro and HOK-hTERT was obtained from Dr. No Hee Park with the help of Dr. David Crowe. Both were cultured in Keratinocyte SFM or Dermalife K Medium Lifeline Cell Technology. All cells were plated and then total cell RNA harvested at 48 hours with cells 50 -80% confluent in a 6 cm dish using the same methods applied to cells from brush cytology. Quantification using RT-qPCR was as described in methods sections by measuring miR-23a, miR-30c and miR-191 as internal controls for RNA level (Exiqon, Woburn, MA).

Supplemental Figure 1 Levels of miR-503-5p and miR-196a-5p *in vivo* and *in vitro* In epithelium differ. A. Relative expression levels of miR-503-5p in brush cytology harvested normal and cancer epithelium were compared using qRT-PCR after normalization for expression of three miRNAs . A similar comparison among epithelial cells in culture, primary oral keratinocyte strains, 1-1 and 1-2, nonmalignant oral cell lines, immortalized by various means, HOK-hTERT and TIGK and 4 OSCC lines, CA9-22, HSC-2, HSC-3, HSC-4 was done. B. The same as above for miR-196a-5p.
